# Supramolecular aggregation of aquaporin-4 shapes astrocyte collective migration and mechanics

**DOI:** 10.1038/s41598-026-35900-z

**Published:** 2026-01-22

**Authors:** Barbara Barile, Nicholas John Mennona, Maria Grazia Mola, Alessia Memeo, Antonio Cibelli, Roberto Barbaro, Matilde Colella, Antonio Frigeri, Valentina Benfenati, Wolfgang Losert, Grazia Paola Nicchia

**Affiliations:** 1 Department of Biosciences, Biotechnology and Environment, University of Bari Aldo Moro, Bari, Italy; 2 Institute for Physical Science and Technology, University of Maryland, College Park, MD USA; 3 Physics Department, University of Maryland, College Park, MD USA; 4 Department of Translational Biomedicine and Neuroscience School of Medicine, University of Bari Aldo Moro, Bari, Italy; 5 Institute of Organic Synthesis and Photoreactivity, National Research Council of Italy, Bologna, Italy; 6 Present Address: Department of Medicine and Surgery, Libera Università Mediterranea “Giuseppe Degennaro”, Casamassima, Italy

**Keywords:** AQP4, Orthogonal arrays of particles (OAPs), Collective cell migration, Biomechanics, Inflammation, Astrocytes, Cell biology, Neuroscience

## Abstract

Aquaporin-4 (AQP4), the main astrocytic water channel, assembles into supramolecular structures known as Orthogonal Arrays of Particles (OAPs). While AQP4-mediated water fluxes are known to influence cytoskeletal dynamics at the single-cell level, their role in collective migration remain unclear. Using primary WT and OAP-deficient (OAP-null) astrocytes, we investigated cell migration under control and proinflammatory conditions. In wound healing assays, control OAP-null astrocytes exhibited greater migratory capacity, while inflamed cells were largely immotile. Particle Image Velocimetry revealed genotype and condition-specific migration patterns, with OAP-null astrocytes displaying more linear trajectories. Cytokine treatment also reduced AQP4 and Connexin-43 expression and impaired gap-junctional communication in both genotypes. Calcein-AM quenching assays showed reduced water permeability in OAP-null cells, independently of treatment. These findings highlight the supramolecular organization of AQP4 as a key determinant of astrocyte collective migration and suggest that chronic inflammation induces a poorly coordinated phenotype through dysregulation of AQP4 and gap-junctional networks.

**Supplementary Information:**

The online version contains supplementary material available at 10.1038/s41598-026-35900-z.

## Main Points

1. The disassembly of AQP4-OAPs enhances the collective behavior of migrating astrocytes by reducing the intercellular repulsion within the cellular bulks.
2. The chronic pro-inflammatory stimulus dramatically impairs the directionality of the motion.

**Supplementary Information:**

The online version contains supplementary material available at 10.1038/s41598-026-35900-z.

## Introduction

Astrocytes migration is reported to occur under physiological conditions during brain development to ensure the maturation of neural networks^1–3^ or in pathophysiological states, like in invasive cancer diseases^4^, spinal cord injury (SCI), or traumatic brain injury (TBI)^5–7^, although more recent evidence indicates that, in response to focal TBI, astrocytes primarily proliferate rather than migrate to form a dense glial border around the site of injury^8,9^.

Cell migration can be defined as a directed movement of a single cell or a group of cells in response to different stimuli, like chemo-attractants^10^, chemokines^2^, or extracellular matrix (ECM) components binding to membrane receptors (i.e. α_v_β_3_ Integrin and Syndecan-4 receptors^11^). Among the plethora of stimuli, it is proposed that water flows can “trigger” cell migration by generating mechanical forces that deform the plasma membrane and sustain the formation of cell protrusions, like lamellipodia and filopodia essential for moving, sensing, and propagating across the environment^6,12,13^. In migrating cells, the depolymerization and polymerization of actin filaments at the leading edge increase the concentration of free ATP-bound G-actin monomers, which in turn raise osmotic pressure and promote water influx^14^.

Aquaporins (AQPs) facilitate this process by increasing water permeability across the membrane, enabling rapid and localized water flux, as widely reported for AQP9 in HEK-293 cells^15^, AQP1 and AQP5 in different types of cancer cells^16,17^, AQP2 in renal epithelial cells^18,19^, and AQP4 in astrocytes^6,12^.

Aquaporin-4 (AQP4) is the most abundant water channel in the brain^20^. Differently from other AQPs, AQP4 is expressed in the plasma membrane of astrocytes as aggregates of tetramers, which form supramolecular crystalline assemblies called Orthogonal Arrays of Particles (OAPs) visible through freeze-fracture electron microscopy (FFEM)^21,22^. This unique ability arises from the selective co-expression in vivo of M23-AQP4 and M1-AQP4 isoforms, that are translated from distinct initiation methionines—M1 (323 aa) and M23 (301 aa)— and, respectively, promote or hinder the aggregation of AQP4 tetramers^23^. The functional and physiological significance of the supramolecular aggregation of AQP4 has not been fully deciphered yet. However, previous studies have hypothesized distinct roles, including ensuring normal AQP4 expression levels and polarization^24^, modulation of synaptic activity^25^ and single-cell migration dynamics^26,27^. The tetramer-forming isoform (M1-AQP4) is proposed to facilitate lamellipodia expansion, while the OAP-forming isoform (M23-AQP4) to enhance cellular adhesivity^26^ and apoptosis, and reduce the invasiveness potential of cancer cells^27^. Of note, also the recently discovered AQP4ex isoform appears to have a crucial role in the supramolecular organization of AQP4 into OAPs, specifically in their formation, stability and proper localization at the astrocytes end-feet^28^.

While delineating the significance of aquaporin channels and AQP4 isoforms as fundamental regulators of individual cell motility, the atomistic-single-cell approach predominant in literature so far led to unaddressed gaps in our understanding of the role of aquaporins in *collective* cell migration. By collective cell migration, we refer to a coordinated movement of cell groups, sheets, or chains^29^. The study of this phenomenon defines how cells interact within a monolayer to orchestrate a single final migratory response^30^, reflecting more accurately what happens in vivo. Collective cell migration is regulated by several mechanisms including an efficient transmission of mechanical propelling forces, the establishment of proper cell polarity and cell–cell junctions, or the contact inhibition of locomotion^31,32^. It has also been demonstrated that the intrinsic physical properties of the extracellular matrix, such as micropatterning^33^ or stiffness^29^ might serve as collective guidance of cell polarization and migration. Accordingly, previous work identified that the use of nanostructured substrates could modify AQP4 permeability, cytoskeletal dynamics, ionic permeability of astrocytes and cell volume regulation^13,34,35^, effects which are accompanied by alteration in cell differentiation. Nevertheless, molecular mechanisms underlying the directionality or the mechanical coupling of the collective motion of cells remain largely unknown.

Although the role of AQP4 in the mechanobiology of astrocytes and glial progenitor cells is well established, its involvement at the collective level remains unexplored^3^. Hence, the present work aimed at assessing whether AQP4 and its supra-molecular aggregation state act as biomechanical regulators of collective cell migration. For this purpose, we investigated the migration of primary cultured brain astrocytes isolated from the Crispr/Cas9-generated OAP-null mice model^24^, genetically lacking the possibility to form OAPs due to the lack of M23-AQP4 isoform, from molecular, biophysical, and dynamical perspectives at the collective cell level. Furthermore, to simulate the pathophysiological context in which astrocyte migration occurs in vivo, we mimicked chronic and severe injury. For this aim, experiments were conducted under control and reactive conditions, where astrocytes were exposed to the major damage-associated pro-inflammatory cytokines, IL-1β and TNF-α.

## Methods

*Animals*: WT and OAP-null pups with a C57BL/6 genetic background were used for primary astrocyte cultures. Procedures involving animals complied with the European and Italian laws on animal use for research and animal care. This project has been approved by the Italian Health Department (F2AEC.N.ETU). The mice used for this study were bred in the approved facility at the University of Bari Aldo Moro. Mice were kept under a 12-h dark-to-light cycle, at constant room temperature (RT) and humidity (22 °C ± 2 °C, 75%). The experiments were designed to minimize the number of animals used and animal suffering. The study is reported in accordance with ARRIVE guidelines.

*Astrocytes Primary Cultures*: Mouse astrocyte primary cultures were prepared from newborn pups (P1–P3), as previously described^36^. Cells were cultured in DMEM High Glucose medium supplemented 10% heat-inactivated fetal bovine serum (FBS), 100 U/mL penicillin, and 100 mg/mL streptomycin and maintained at 37 °C in a 5% CO_2_ incubator. Cell culture reagents were purchased from Euroclone®.

*Pro-inflammatory cytokines treatment*: After 24 h from cell plating, the control medium was replaced with growth medium supplemented with 10 ng/mL TNFα (50,349-MNAE-5) and 10 ng/mL IL-1β (50,101-MNAE-5) (Sino Biological) and replaced every two days for seven days of treatment.

*Immunofluorescence*: Astrocytes were plated on coverslips, fixed in 4% paraformaldehyde, washed in phosphate-buffered saline (PBS), and permeabilized with 0.3% Triton X-100 in PBS. After blocking with 2% BSA in PBS (PBS-BSA), cells were incubated with primary antibodies for 1 h at RT. After rinsing, cells were incubated with Alexa-conjugated secondary antibodies for 1 h at RT. After rinsing, coverslips were mounted using Prolong mounting medium (Thermo Fisher) and examined by confocal microscopy or gated STED (Leica TCS SP8). Post-processing was applied to the images using Adobe Photoshop to create a more accurate tonal and color correction. Protruding filopodia were quantified from inset images of the cell edge, using actin immunostaining captured at 100 × magnification with an additional 5 × digital zoom to improve resolution of the processes. Prior to analysis, images underwent background subtraction and a 2-pixel Gaussian blur filtering. For each cell inset, the length of each filopodia emerging from the cell boundary was measured. The total number of filopodia was then counted and expressed as the number normalized to a 50 µm membrane perimeter, allowing comparison across cellular edges of different sizes.

*STED super-resolution imaging*: Leica TCS SP8 STED 3 × microscope and Leica LASX software were used for image acquisition and analysis (Leica Microsystems CMS GmbH). A Leica HC PL APO 100x/1.40 Oil STED White objective and Type F Immersion liquid with a refractive index of 1.5 were used. Excitation of the Alexa Fluor488 dye was achieved with a continuous wave at 488 nm using a diode laser with a maximum light output in the focal plane of 10 mW (NKT Photonics supercontinuum laser). Depletion was performed using a fiber laser with a continuous wave of 592 nm and a maximum light output in the focal plane of < 0.5 mW (Laser Quantum). For Cx43, multiple regions of 20 µm^2^ were imaged within cell-to-cell regions. STED images were deconvoluted in ™Huygens Software and segmented in ImageJ using Trainable Weka Segmentation.

*Western Blot Analysis*: 2 × 10^5^ cells were plated onto PDL-coated dish (35 mm diameter). As previously reported^36^, cells were solubilized in RIPA buffer (25 mM Tris–HCl, pH 7,6; 150 mM NaCl; 1% Triton X-100; 1% sodium deoxycholate; 0,1% SDS) added with a cocktail of protease inhibitors. The lysis was performed on ice for 30 min and the samples were then centrifuged at 21,000 g for 30 min at 4 °C. The protein content of the supernatant was measured with a bicinchoninic acid (BCA) Protein Assay Kit (Thermo Fisher). 10 µg of protein samples were loaded into 13% polyacrylamide gel (for resolving AQP4 isoforms in WT astrocytes), 12% (for M1-AQP4 in OAP-null astrocytes) and 10% TGX Stain-Free FastCast gels (Biorad) (for GFAP and Cx43) and transferred to polyvinylidene fluoride (PVDF) membranes (Biorad). Membranes with blotted proteins were incubated with primary antibodies, washed, and incubated with peroxidase-conjugated secondary antibodies. Proteins were revealed with an enhanced chemiluminescent detection system and visualized on a Chemidoc imaging system (Biorad). Densitometric analysis was performed with Image Lab software by using Stain-Free staining of total protein as a normalization channel (for GFAP, Cx43, and M1-AQP4) and GAPDH (for M1-AQP4 and M23-AQP4 in WT astrocytes).

*Antibodies and fluorescent dyes*: The following primary antibodies were used for Western Blot and immunofluorescence: rabbit anti-AQP4 (Sigma, diluted 1:500); rabbit anti-Cx43 (Sigma, diluted 1:1000); mouse anti-GFAP (Sigma, diluted 1:1000), mouse GAPDH (Abcam, diluted 1:1000). The following secondary antibodies were used: Horseradish Peroxidase (HRP) conjugated donkey anti-rabbit antibody (diluted 1:5000) for Western blot; donkey anti-rabbit IgG-Alexa Fluor™ 488 (Thermo Fisher, diluted 1:1000), goat anti-mouse Alexa Fluor™ 594 (Thermo Fisher, diluted 1:1000), and Alexa Fluor™ 647 Phalloidin (Thermo Fisher, diluted 1:1000) for immunofluorescence.

*Fluorescence-quenching water transport assay*: Cells were seeded on black, clear-bottom 96-well-plates (Corning, NY) at a density of 12,000 cells per well as previously described^34^ and analyzed 7 days after plating under control and proinflammatory conditions. Cells were loaded with 10 μM Calcein-AM at 37 °C for 20 min in growth medium and then rinsed in isotonic PBS. The principle behind the technique^37^ and the related implications in tracking cell volume changes have been previously described^36,38^. Fluorescence signal changes were recorded on a Flex Station3 plate reader equipped with an integrated automatic liquid handling module (Molecular Devices). The hypotonic challenge was induced by adding an appropriate volume of water to obtain a 60 mOsm/L osmotic gradient. The fluorescence signal increases following the hypotonic stimulus due to cell swelling. Data acquisition was performed using SoftMaxPro software and the data were analyzed with Prism (Graph Pad 8) software. The time constants τ (s) of cell volume variation upon swelling phase were obtained by fitting the data with an exponential function. Calcein fluorescence of representative kinetics are expressed as fluorescence ratio F/F0 in Arbitrary Units (A.U.) of fluorescence intensity. The spans are measured as the difference between Y_max_ following the hypotonic shock and Y_min_ before the hypotonic shock.

*Wound healing assay*: Wound healing assay was conducted on confluent astrocyte monolayers to assess their migratory ability in response to injury, by measuring their ability to repopulate the scratched area**.** 1 × 10^5^ cells were plated onto PDL-coated confocal dishes (35 mm diameter) and evenly distributed on the inner growing area. After 7 days of culture in 10% FBS growth medium, 100% confluent monolayers were incubated in CTRL or cytokine-enriched media both supplemented with 1% FBS for an additional 24 h before imaging to minimize potential effects of cell proliferation, as previously reported^39^. On the day of imaging, two parallel wounds were manually created on the confocal dish with a P10 tip and spaced to align with the edges of a 6 × 6 mm imaging area. Migrating cells were imaged for 24 h in CTRL or cytokine-enriched media, each supplemented with 1% FBS, using a BioStation IM-Q (Nikon). For the analysis, the free gap between the two cellular fronts was measured at 0, 6, 12, 24 h in ImageJ. Wound healing ability was expressed as percentages (%) of the wounded area, with the total (100%) being the initial area of the wound. The average analyzed scratch dimension was 43,872 µm^2^ ± 598 SEM for WT CTRL, 42,816 µm^2^ ± 560 SEM for OAP-null CTRL, 42,792 µm^2^ ± 578 SEM for IL-1β/TNFα-treated WT, and 43,908 µm^2^ ± 517 SEM for IL-1β/TNFα-treated OAP-null. The mean scratch size was not statistically different among conditions and genotypes (Kruskal–Wallis test, *p* > 0.05*)*, thus ensuring the same initial wound dimension. The number (*n*) reported in the figure legends refers to the fields imaged and included in the final analysis, obtained from two wounds per dish of three independent biological replicates (N). The effects of FBS supplementation on wound-healing dynamics were further evaluated through additional scratch-assay experiments (consult Supplementary materials, Figure S1).

*PIV analysis*: The computer vision algorithm PIV^40^ was applied to the astrocyte wound healing dataset to identify the migratory phenotypes of the genotypes under the aforementioned experimental conditions. The patterns that were taken into account were the directionality (i.e., the ability of the cell sheet in moving *forward* to close the wound), the speed (i.e., how fast cells close the wound), and strain (i.e., whether the path of the wound closing is straight or erratic). These mechanical patterns were quantified through Particle Image Velocimetry (PIV)^41^, which tracks the bulk movement within a certain region (ROI) of an image sequence across time. PIV quantifies the motion of bulk sheets by dividing the sheets into smaller areas (sub-regions) and identifying similar motion between two frames (separated by some time interval) within paired sub-regions. These sub-regions are then cross-correlated to ascertain the displacement (or motion) occurring in this area of the sheet. A comprehensive, technical overview of PIV can be found in the work from Scharnowski and Kähler^42^. For a comprehensive technical overview of decomposing kymographs into features for dimensionality reduction, consult Zabary^40^. Our dataset comprises bright field imaging; the frame size is 600 × 800 pixels, and each time-lapse lasts 24 h. Due to the migratory nature of the data, we apply Particle Image Velocimetry (PIV) to the dataset. We use initial windows of 48 × 48 pixels with subsequent interrogation windows of 24 × 24 with 50% overlap. These parameters were selected based on manual assessment of sheet migration over time. Since the data collected is from the brightfield channel, other methods of segmentation/boundary location usually applied in comparable studies is out of the reach of this analysis. PIV outputs data matrices containing velocity vectors in predetermined points in the image frame; from an image matrix of 600 × 800, given the above parameters, a PIV output frame contains 49 × 65 pixels. Each location (corresponding to the center of the block PIV pixels) contains a velocity vector corresponding to frame motion. PIV is a method for correlating the motion vectors (matching image features) in small windows in a dataset across time. In order to use PIV, we manually estimate motion in an astrocytic sheet (Fig. 3a). We use our estimates of both boundary and bulk motion to determine proper parameters for PIV estimation. For this data, we use initial windows of 48 × 48 pixels with subsequent interrogation windows of 24 × 24 with 50% overlap. As an example, from our image matrices of 600 × 800, a PIV output frame contains 49 × 65 pixels. A small window of PIV output is shown. The PIV vectors provide us with the ability to measure (1) total distances travelled (time average), (2) velocity kymographs (y-axis average), and (3) strain kymographs (y-axis average). These metrics allow us to classify the dynamical patterns seen in migratory astrocytes. Each pixel in kymograph corresponds to its distance away from the frame edge and its value in time; we take the median velocity of the column a certain distance away from the edge to compute that pixel in the kymograph.

For the time average calculation (visualization generated by summing the output PIV frames and dividing by frame number), we find that OAP and reactive astrocytes respond idiosyncratically to a wound. In other words, the distributions (Fig. 3d) for OAP-null CTRL are more positive (migration toward the wound), IL-1β/TNFα-treated OAP-null are more negative (migration away from wound site), WT CTRL are centered around zero, and IL-1β/TNFα-treated WT are similarly centered around zero, but contain a larger variance in activity. We further probe these distributions by examining the kymographs of velocity (the subset of the time average) and strain. In order to assess the shared dynamics, we take the kymographs and divide them into chunks; from these ‘chunks’ we take the summation of the entire chunks. Across all chunks we now have generated a vector with reduced data size; for example, assume we generated a kymograph of size 32 × 144. We divide this matrix into 8 distinct chunks. We would then have a feature vector (size 8 × 1) which retains properties of each kymograph for population-wise comparison. These feature vectors and dimensionality reduction to quantify and classify the dynamical patterns seen in migratory astrocytes.

*Scrape loading / Lucifer-Yellow (LY) assay*: 1 × 10^5^ cells were plated in PDL-coated confocal dishes (35 mm) and evenly distributed on the inner growing area. After 7 days, the cell culture medium was removed and 400 µL of 1 mg/mL Lucifer Yellow dissolved in PBS were added onto 100% confluent monolayers. Cx43 shows high permeability for molecules with molecular weight above 400 Da, such as LY dye which cannot freely permeate the plasma membranes of the cells unless they are broken or, as in the case of the scrape loading assay, voluntarily damaged. Cells monolayers were, therefore, manually scratched with a scalpel blade and incubated with LY in the dark at RT for 10 min to let the dye get into the damaged cells and diffuse across cell bulks depending on Cx43 levels. The dye was discarded and stored at 4 °C for further use, then the cells were thoroughly rinsed in PBS twice, fixed for 10 min in 10% PFA, and rinsed again. Fixed samples were analyzed right after using a Nikon Eclipse TE2000-U inverted microscope, equipped with 10X Plan Fluor objective, DeltaRAM V high-speed random access monochromator, and photometrics CCD CoolSNAP camera. Images were acquired with the imaging software Metafluor (acquisition parameters: binning 1, λexc: 388 nm; λem: 530 nm). An average of 8 different images per scratch (3 scratches per technical replicate) were acquired along the monolayer wounds keeping orientation and position constant among the acquisitions. To record the fluorescence background of the monolayer, three images were acquired far from the wounds as an internal negative control for each sample. Image analysis was performed on ImageJ measuring the fluorescent area as an indicator of the dye spreading and, consequently, the gap junctional connectivity of the cells.

### Experimental design and statistical analysis

All data represent replicates from biologically independent experiments. The number of independent experiments (N) and technical replicates (*n*) is provided in the figure legends. Different letters located on top of the bar indicate a significant difference (*p* < 0.05) while equal letters indicate no significant difference (*p* > 0.05). In the box plots, each box represents the interquartile range (IQR), the central line the median, and the plus sign “ + ” the mean value. The whiskers extend from the minimum to the maximum values. Statistical analysis was performed in GraphPad8 software. Statistical significance was evaluated using the statistical test indicated in each figure legend. *p*-values are defined as follows: *p < 0.05, **p < 0.01, and ***p < 0.001 and ****p < 0.0001.

## Results

### AQP4 aggregation state does not impact astrocyte response induced by IL-1β and TNF-α treatment

**Fig. 1:**
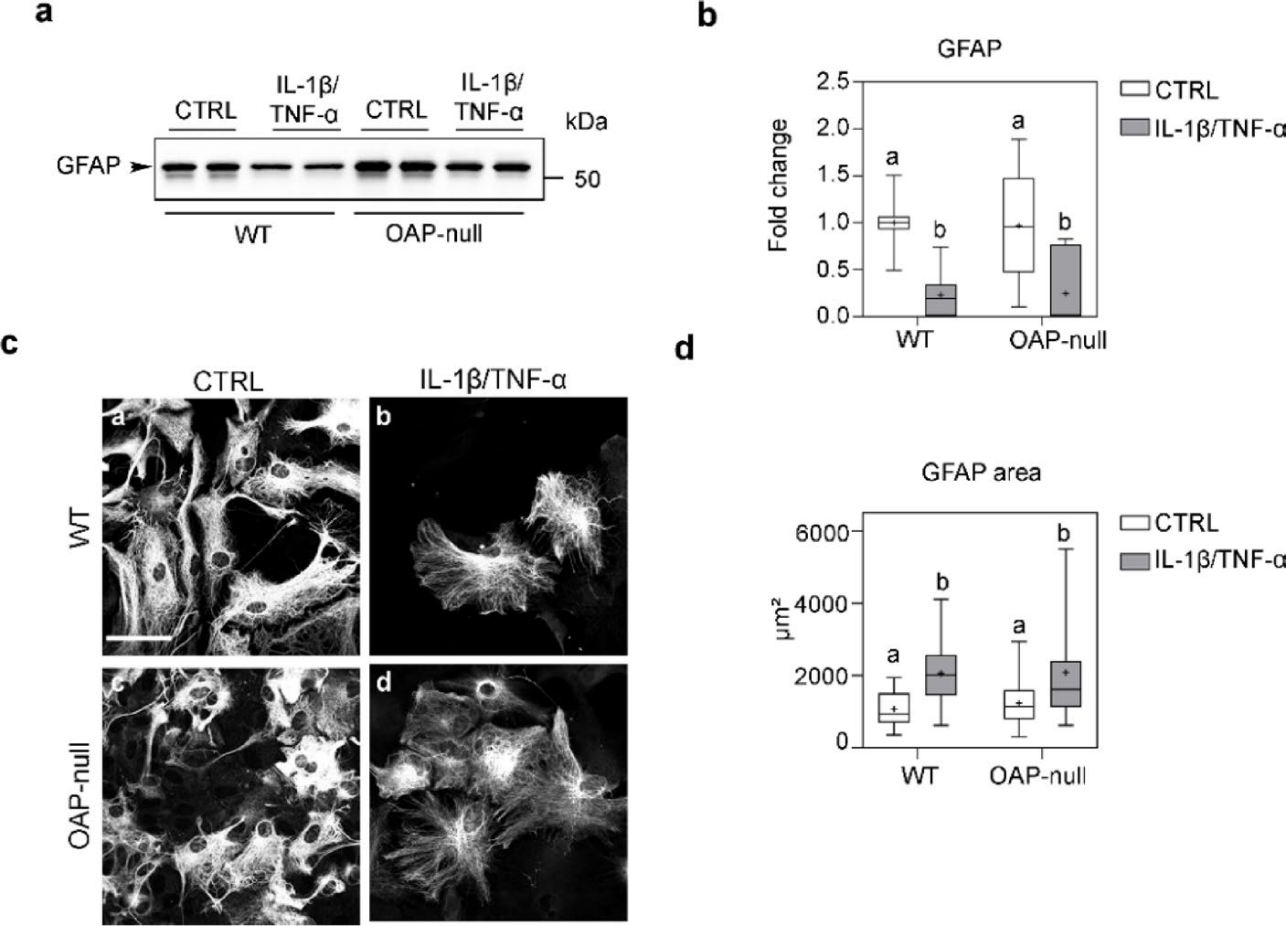
Characterization of the i*n vitro* model of astrogliosis upon IL-1β/TNF-α exposure. Western Blot (**a**) and densitometric (**b**) analysis of GFAP expression normalized against total protein. Two-way ANOVA with Tukey’s Multiple Comparison test, with *n* = 10 for each group from N = 5 independent experimental dataset. Refer to Table S1 for details on significant differences between groups and related *p*-values and Figure S2 for uncropped western blot of GFAP. (**c**) Confocal images of GFAP staining (scale bar: 50 μm). (**d**) Box plot of morphometric analysis based on GFAP positive areas showing an increased area in IL-1β/TNF-α-treated astrocytes compared to controls. Two-way ANOVA with Tukey’s Multiple Comparison test was performed on 10–38 fields, with *n* ranging from 25 to 41 cells from N = 3 independent experimental dataset. Consult Table S2 for details on significant differences between groups and related *p-*values.

To validate the model of chronic reactive gliosis, we determined astrocyte activation following prolonged exposure (seven days) to IL-1β and TNF-α. WT and OAP-null astrocyte responses were assessed through the evaluation of GFAP expression levels and the GFAP surface area of astrocytes (Fig. 1).

Quantitative analysis revealed a significant downregulation of GFAP protein expression in both IL-1β/TNF-α-treated WT and OAP-null astrocytes (Fig. 1a, b) while morphometric analysis based on GFAP immunostaining^43,44^ demonstrated a notable increase in the surface area occupied by intermediate filaments (Fig. 1c, d) compared to untreated control (CTRL) cells. No differences were observed between genotypes under the same conditions, indicating that the AQP4 aggregation state does not influence astrocyte reactivity in response to the inflammatory stimulation.

### AQP4-aggregation state influences astrocyte migratory ability which is impaired by IL-1β/TNF-α treatment

**Fig. 2:**
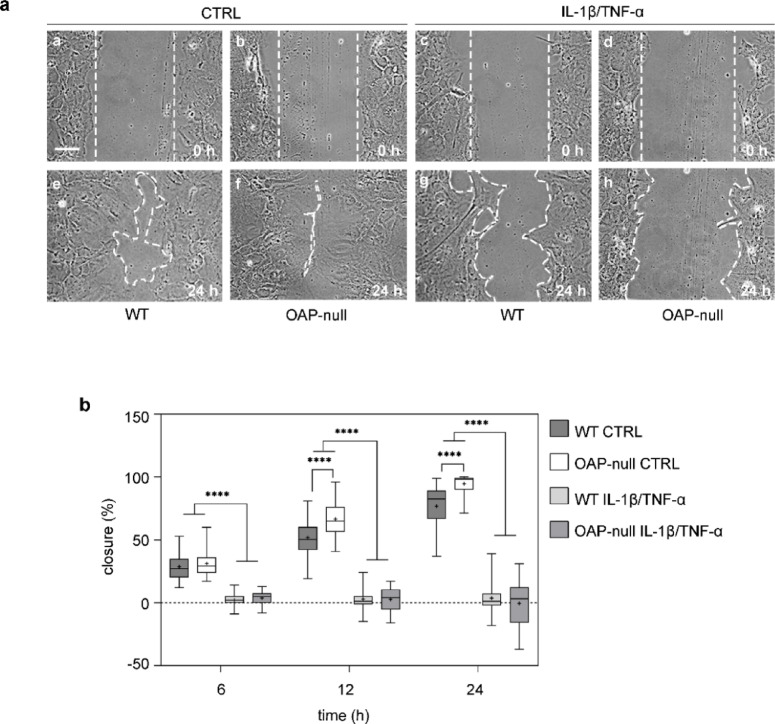
Wound healing assay analysis. (**a**) Representative phase contrast images of cell sheets injured with a wound of ~ 200 µm in length for all conditions at 0 (A-D) and 24 h (E–H) (scale bar: 50 µm). (**b**) Box plots showing wound healing percentages of the scratch wound closure (%). At 6 h, no differences were found between genotypes, but between controls compared to IL-1β/TNF-α-treated astrocytes (****p < 0.0001). At 12 and 24 h a significant difference was found between WT and OAP-null cells under CTRL conditions (****p < 0.0001) and between CTRL astrocytes and treated ones (****p < 0.0001). At each time point (6; 12; 24 h) Two-way ANOVA with Tukey’s Multiple Comparison test was performed on the mean values across the four groups with *n* ranging from 42 to 51 fields per group and from N = 3 independent experimental datasets.

We next evaluated collective astrocyte migration using a wound healing assay (Fig. 2a, b).

Under control conditions, OAP-null astrocytes exhibited a significantly greater wound closure compared to WT, almost completely sealing the scratch within 24 h. In contrast, IL-1β/TNF-α treatment, severely impaired migration in both genotypes with astrocytes remaining almost immotile and with OAP-null astrocytes occasionally expanding the wound area.

Overall, these results show that the AQP4-aggregation state plays a crucial role in regulating astrocyte migration under normal conditions, with AQP4-tetramers further enhancing the migratory ability of cells compared to AQP4-OAPs.

### Particle image velocimetry (PIV) reveals that AQP4 aggregation state influences collective astrocyte migration patterns

To further characterize the collective dynamics of astrocyte sheets during wound healing, we used Particle Image Velocimetry (PIV)^41^ analysis. In particular, the PIV computer vision algorithm^40^ was applied to the astrocyte wound healing dataset to quantify motion parameters such as directionality, speed and strain.

**Fig. 3:**
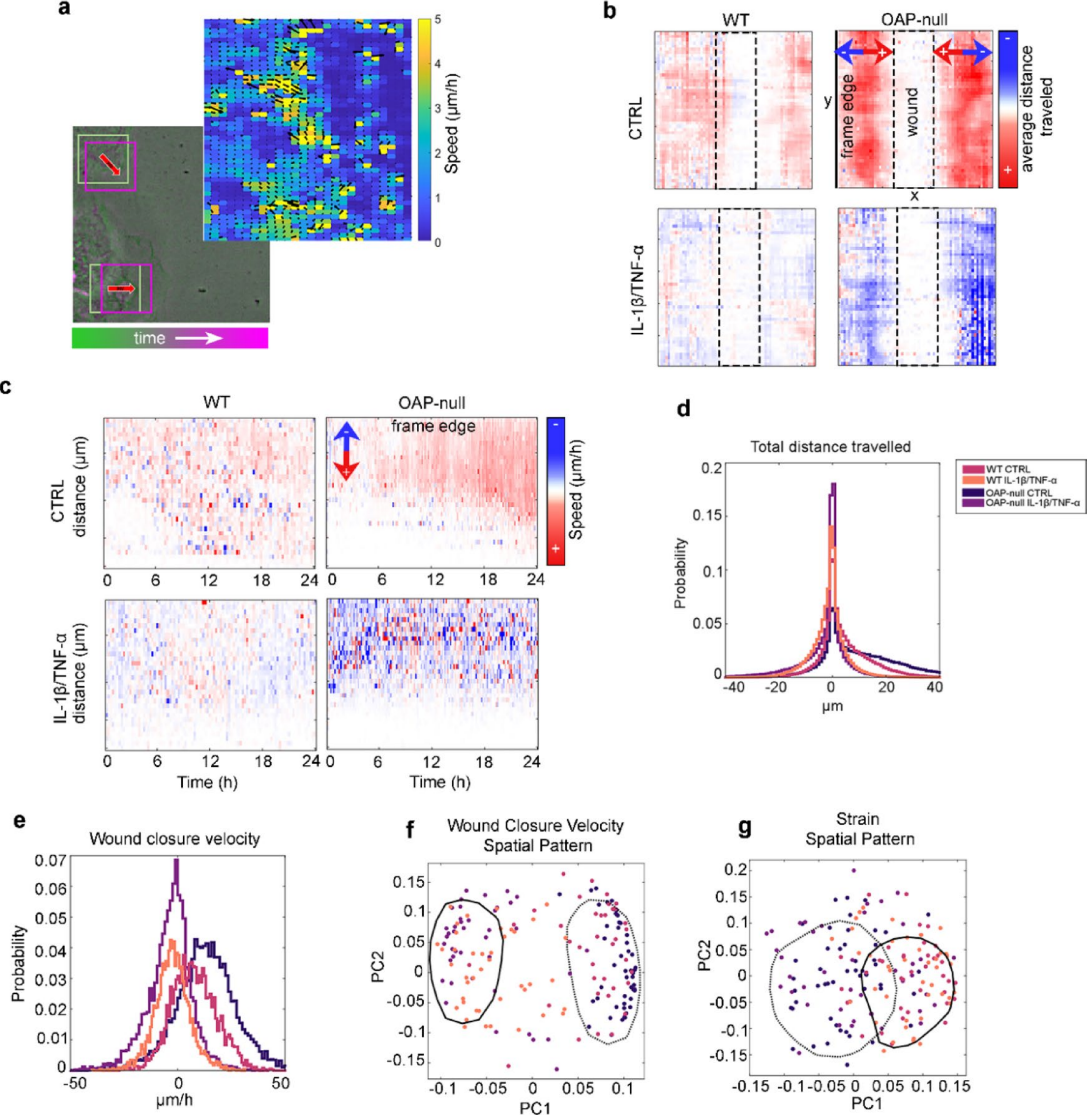
PIV used to distinguish spatiotemporal dynamics differences in OAP and WT astrocyte migrating sheets. (**a**) PIV schematic. PIV detection of bulk movement frame to frame. Window size used is 48 × 48 pixels down-sampled by 50% for secondary matching. Representative PIV vector field. Scale bar on order of µm/min. The PIV analysis was performed on the wound-healing time-lapse recordings. The numbers of fields (n) and independent experiments (N) match those reported in Fig. 2. (**b**) ‘Summation maps’ of horizontal velocity PIV. Representative samples for the four conditions are shown, indicating positive (in red) and negative (in blue) wound healing. (**c**) Representative kymographs indicating average speed at different distances from the wound site (middle of frame) as a function of time. Red indicates movement toward the wound site, blue movement away from the wound. (**d**) Histogram of summed displacements toward the wound site. (**e**) Histogram of wound closure speed (mean speed/frame in image sequences. (**f**) PCA dimensionality reduction on time averaged speed spatial pattern from (c). The analysis highlights the existence of two different clusters based on reactivity instead of the AQP4-aggregation state. (**g**) PCA dimensionality reduction on strain spatial pattern revealing separation into two different clusters according to AQP4-aggregation state instead of reactivity.

The PIV pipeline illustrating the matching windows (overlapping squares) and representative vector field (arrows) is shown in Fig. 3a.

In our dataset, the expected direction of motion was horizontal as both sheets move toward the wound from opposite sides. Therefore, the horizontal velocity (‘wound closure velocity’) and the cellular vorticity (‘strain’) of wound-healing cellular sheets were measured.

The PIV vectors were used to generate summation maps of the horizontal velocity (Fig. 3b). As indicated by the color bar, red pixels correspond to positive horizontal velocities (i.e. migration directed toward the wound), blue pixels to negative horizontal velocities (i.e. backwardly motion), and white pixels to no movement. Representative results for the four experimental conditions are shown in Fig. 3b. Under CTRL condition, OAP-null astrocytes exhibited a predominant forward movement, while IL-1β/ TNF-α-treated OAP-null astrocytes showed a significant shift toward back movements. Conversely, WT astrocytes displayed more heterogeneous patterns with mixed forward and backward motion in both conditions, although IL-1β/ TNF-α-treated WT astrocytes tended to show more backward shifts.

To further analyze the migratory dynamics, we generated kymographs representing the median velocity as a function of distance from the wound over time (Fig. 3c). Principal Component Analysis (PCA) was used to reduce the dimensionality of our feature vectors extracted from the kymographs in order to cluster.

By assessing both horizontal motion and strain, statistically significant differences within the groups were found. Analysis of the wound closure velocity summation maps allowed us to calculate both the total distance traveled by astrocytes (speed * time = distance) (Fig. 3d) and their mean horizontal speed per frame (Fig. 3e). In terms of total distance traveled (Fig. 3d), CTRL OAP-null astrocytes showed distributions skewed toward positive values, indicating effective migration toward the wound. In contrast, IL-1β/TNF-α-treated astrocytes, exhibited distributions shifted toward negative values, indicating backward movement. CTRL WT astrocytes displayed distributions centered around zero, while IL-1β/TNF-α-treated WT astrocytes similarly centered around zero but with increased variance.

More detailed insights were provided by the distributions of the mean horizontal speed for wound closure (Fig. 3e) showing that astrocytes under inflammatory conditions exhibited predominantly null or negative speeds compared to controls, consistent with the impaired or reverse migration observed in Figs. 2a, b and 3b. Of note, OAP-null astrocytes showed the most positive distribution of wound closure velocity, confirming their higher migratory capability under physiological conditions.

PCA of the wound closure velocity featured a clear separation of the experimental datasets into two distinct clusters (Fig. 3f). Clusters are enclosed by distinct contours (as defined by bold or dotted lines), confirming that the patterns of horizontal migration speed are statistically distinct between CTRL and IL-1β/TNF-α -treated astrocytes. These findings are consistent with the different distance and velocity distributions shown in Fig. 3d,e.

In contrast, comparable PCA on the strain kymographs (strain equivalent of the velocity kymographs shown in Fig. 3c) showed that clustering was independent of the inflammatory treatment and instead correlated with the AQP4-aggregation state (Fig. 3g). While OAP-null astrocytes maintained cohesive sheets without shear deformations, WT astrocytes exhibited marked intercellular strain and partial sheet disruption, regardless of treatment.

These results show that AQP4 aggregation state significantly influences the coordination and mechanical dynamics of collective astrocyte migration, promoting more direct and cohesive motion in the absence of OAPs. Moreover, the pro-inflammatory stimulation here used severely impairs these dynamics, overcoming astrocyte migratory properties linked to AQP4 aggregation state.

### AQP4 aggregation state and inflammation shape actin architecture and mechanical coherence at the leading edge of migrating astrocyte sheets

**Fig. 4:**
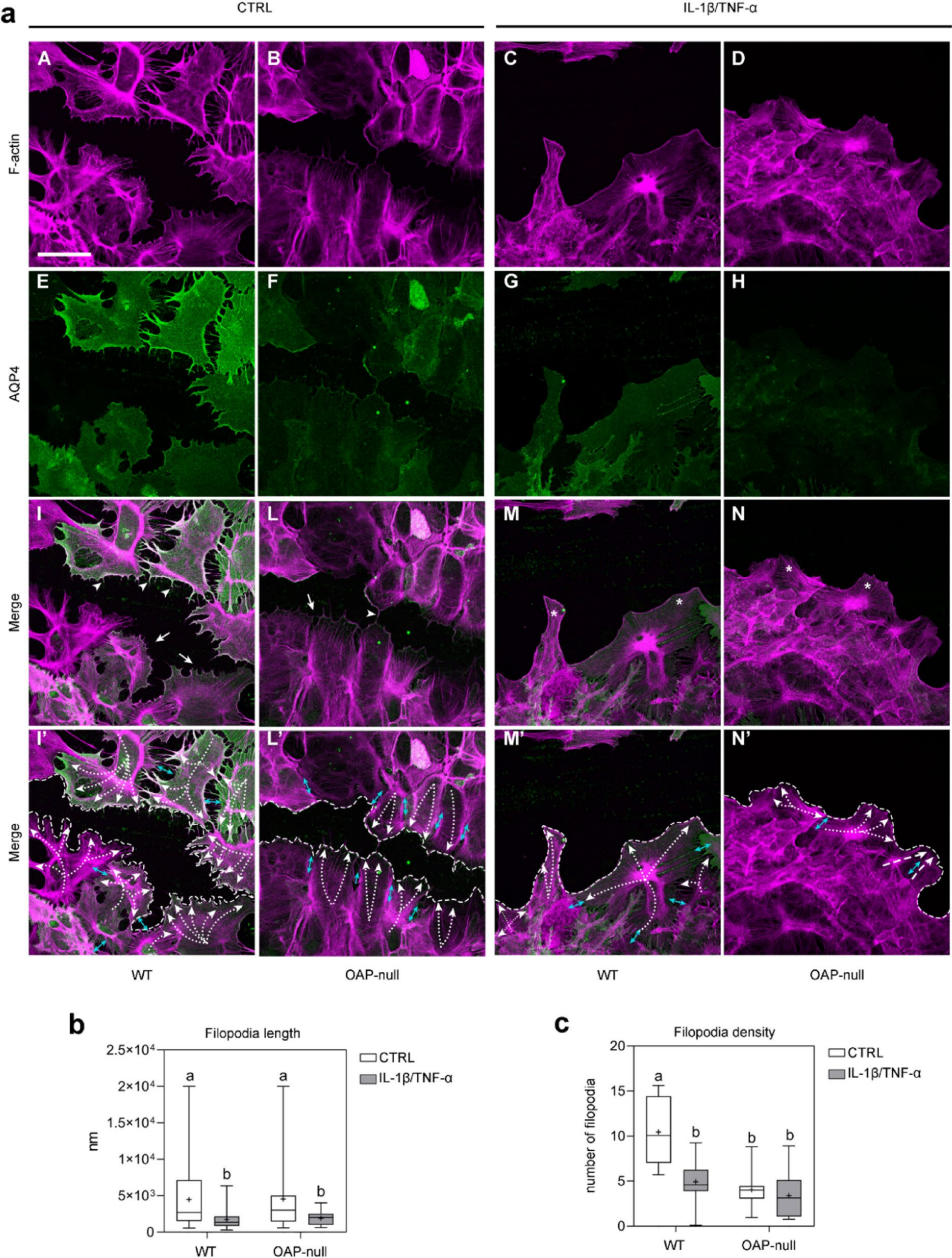
AQP4 and F-actin staining in the follower cells of injured astrocyte sheets. (**a**) Confocal images of F-actin (magenta), AQP4 (green) staining and merged signals in migrating astrocytes at 24 h after the scratch (scale bar: 100 µm) showing the migration front (dashed line), actin cell structures (lamellipodia: white arrows; filopodia-like structures: arrowheads; stress fibers: asterisks), and PIV output (cell strain: cyan double-headed arrows; horizontality: white dashed curved lines). (**b**) Box plots showing the filopodia length. The analysis revealed longer filopodia in CTRL cells compared to treated astrocytes. Two-way ANOVA with Tukey’s Multiple Comparison test was performed on the mean values across the four groups, with *n* ranging from 41 to 68 data points from N = 3 independent experimental datasets. Consult Table S3 for significant differences between groups and related *p-*values. (**c**) Box plots showing the filopodia abundance measured as the number of protruding filopodia normalized to a 50 µm membrane perimeter. Two-way ANOVA with Tukey’s Multiple Comparison test was performed on the mean values across the four groups, with *n* ranging from 12 to 21 cells from N = 3 independent experimental datasets. The analysis highlighted an augmented number of filopodia in WT CTRL astrocytes compared to the other conditions. Refer to Table S4 for significant differences between groups and related *p-*values.

Given that both AQP4 aggregation state and IL-1β/TNF-α treatment significantly impact collective migration dynamics, modulating speed, directionality and mechanical coordination, we later investigated whether the results obtained were associated with changes in actin cytoskeleton at the migrating front (Fig. 4).

WT astrocytes exhibited extended lamellipodia, characterized by a branched actin network at the leading edge, indicative of active membrane remodeling. Filopodia-like structures appeared as fine, pointed protrusions emerging from the lamellipodial border. Of note, WT and OAP-null control astrocytes displayed the longest filopodia among all conditions, indicating that filopodial elongation is largely preserved in untreated cells regardless of OAP presence. However, only WT astrocytes showed the highest number of filopodia compared with all other groups, suggesting that while filopodial length is similarly maintained in both genotypes, WT cells uniquely sustain a more robust filopodial density.

Organized radial actin bundles were visible along the cell periphery, indicating a tense cytoskeletal structure. However, the migration front was irregular with discontinuities between adjacent cells, consistent intercellular strain and mechanically uncoordinated migration.

In contrast, OAP-null astrocytes displayed a smooth and continuous leading front, with reduced isolated lamellipodia. Cortical actin was uniformly distributed along the cell perimeter indicating a stable cytoskeletal architecture. Adjacent cells at the front appeared aligned and cohesive, indicative of a cohesive and mechanically integrated front.

These results show that under physiological condition WT astrocytes show a mesenchymal-like behavior with prominent individual protrusions and irregular border leading to a poorly coordinated front. Conversely, OAP-null astrocytes exhibit an epithelial-like pattern, with uniform actin cortex, structural continuity, and directional coherence, supporting efficient collective migration.

Upon IL-1β/TNF-α-treatment both genotypes displayed a marked loss of protrusive structures required for directional migration, replaced by stiff and thick actin stress fibers arranged in parallel or radial bundles^45^, indicative of impaired migration ability.

In inflamed WT astrocytes, the migration front appeared irregular and disrupted, with a clear loss of cohesion between adjacent cells while in inflamed OAP-null astrocytes, the front appeared more linear and continuous. Cells remained aligned, with a more homogeneous cortical actin distribution, suggesting preserved structural integrity. Here too, migration was virtually absent, as shown by wound closure curves and horizontal velocity profiles; however, unlike in WT, mechanical cohesion was maintained, with no detectable intercellular strain.

These results indicate that inflammatory signals rather than AQP4 aggregation state profoundly alter cytoskeleton architecture in migrating astrocytes with loss of lamellipodia and filopodia-like structures essential for protrusive activity and directional migration.

Moreover, while inflammatory treatment nearly abolishes migratory capacity in both genotypes, the degree of mechanical disorganization still reflects the AQP4 aggregation state.

### Inflammation disrupts cortical actin organization in follower cells without altering AQP4 distribution

**Fig. 5:**
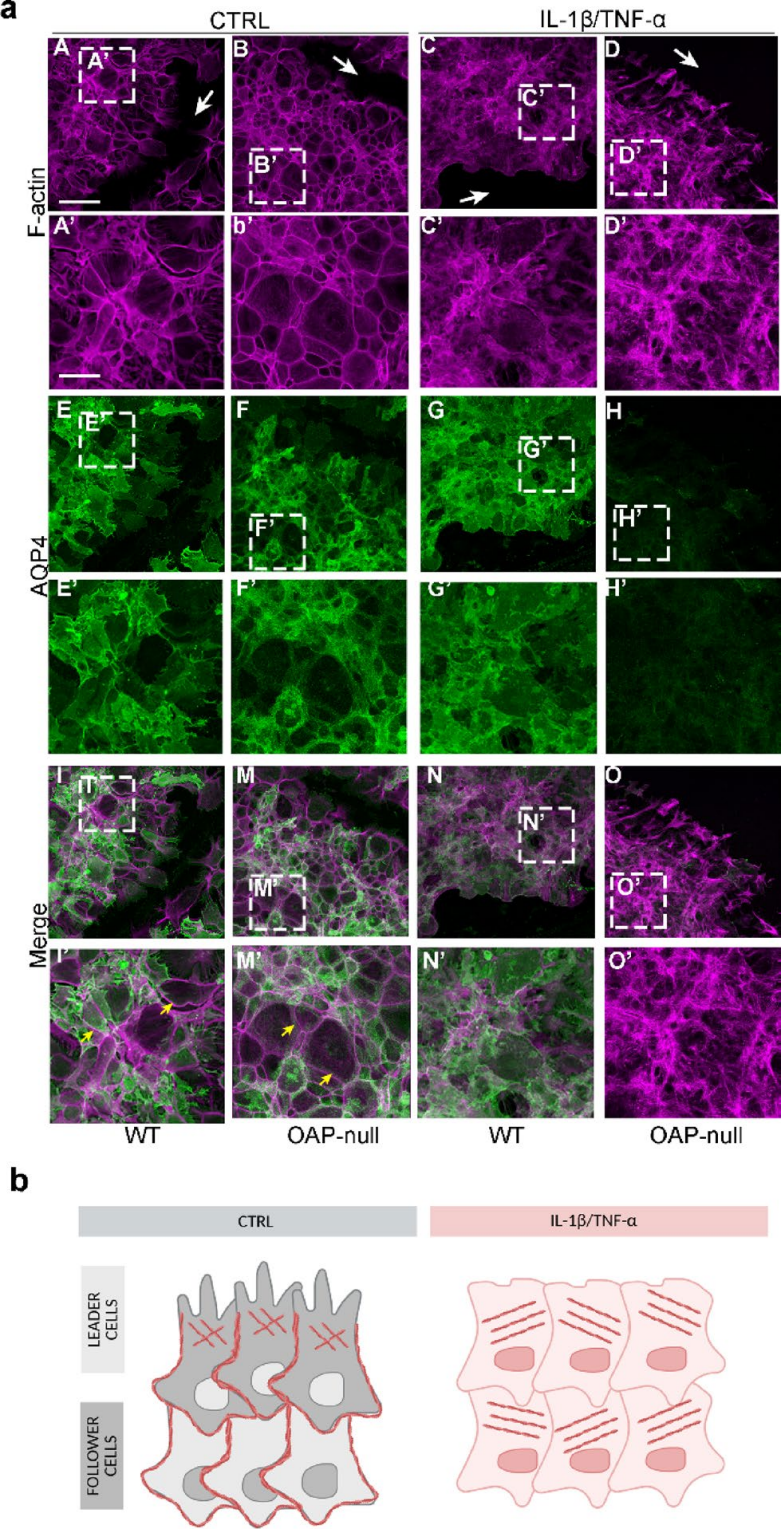
AQP4 and F-actin staining of injured astrocyte sheets. (**a**) Confocal images (a-o; scale bar: 100 µm) with insets in dashed-lined rectangles (a’-o’; scale bar: 50 µm;) of F-actin (magenta), AQP4 (green) staining, and merged signals of follower cells in migrating astrocytes at 24 h after the scratch (cortical actin: yellow arrow). In IL-1β/TNF-α-treated cells, actomyosin network is profoundly disrupted in randomly dispersed cytoplasmic fibers compared to CTRL astrocytes. (**b**) Proposed model of F-actin rearrangements in leader and follower CTRL cells and unpolarized IL-1β/TNF-α-treated cells (Created in BioRender. Barile, B. (2026) https://BioRender.com/pgn5sgv).

To determine whether the cytoskeletal alterations observed at the leading front extend to the follower cells within the migrating sheet, we analyzed the distribution of F-actin and AQP4 under control and inflammatory conditions (Fig. 5).

Under control condition, both WT and OAP-null astrocytes exhibited a well-organized cortical actin pattern, with fibers distributed uniformly along the cell periphery, indicating lateral tension and structural cohesion throughout the monolayer. Upon IL-1β/TNF-α treatment, actin organization appeared markedly altered, similarly in both genotypes, with stress fibers arranged irregularly and a clear loss of cortical actin, suggesting compromised mechanical integrity across adjacent cells. AQP4 immunolabeling revealed no appreciable differences between leader and follower cells or across experimental conditions. These findings suggest that inflammation, rather than AQP4 supramolecular organization, disrupts actin cytoskeleton architecture in follower cells, contributing to the impaired collective migration observed under pro-inflammatory conditions.

### Chronic inflammatory treatment downregulates AQP4 expression without reducing water transport rate

**Fig. 6:**
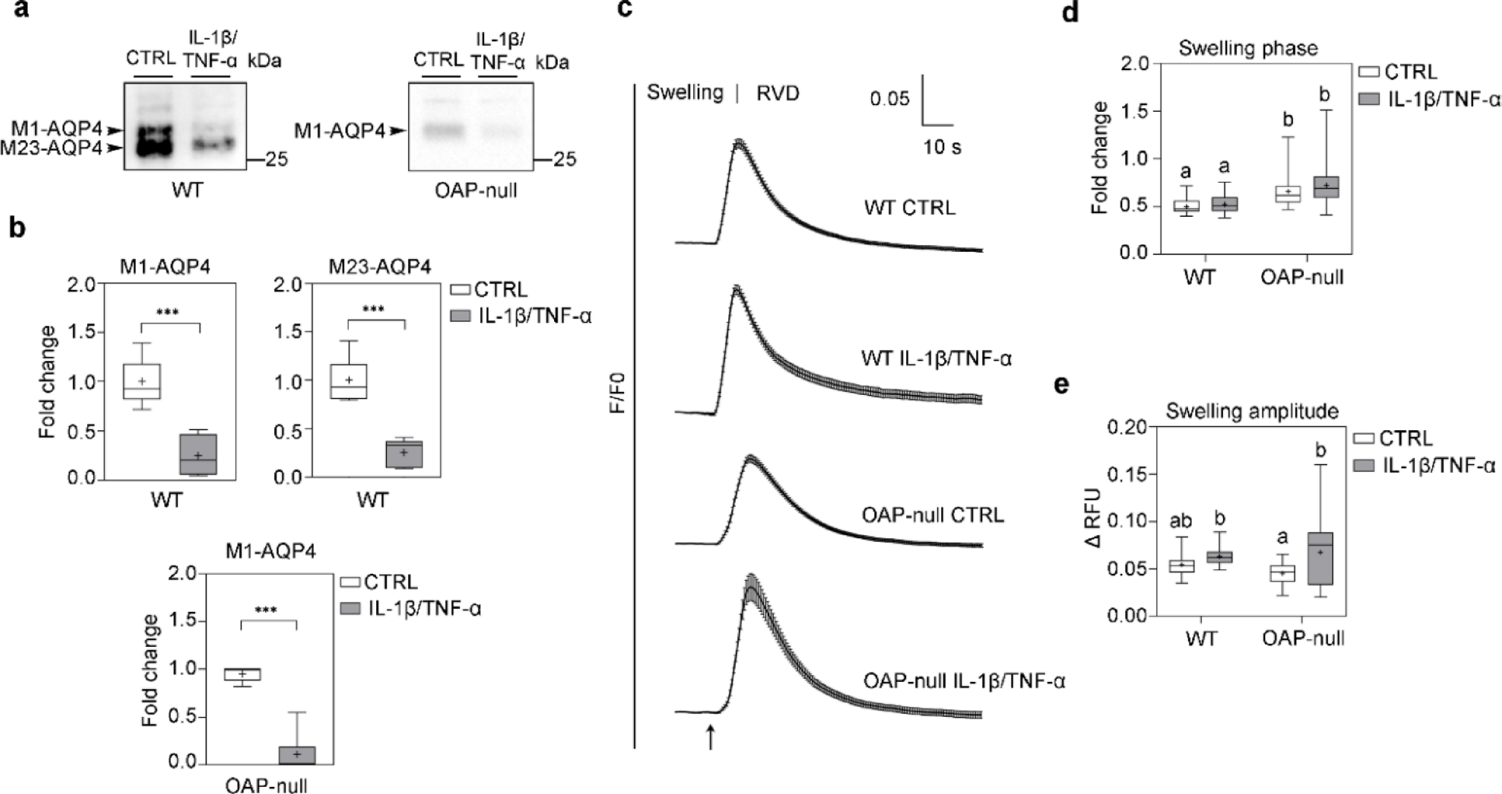
AQP4 expression levels and AQP4-mediated water transport kinetics. (**a**) Western Blot analysis of AQP4 expression in CTRL and IL-1β/TNF-α treated WT and OAP-null astrocytes. AQP4 is revealed as two separated bands at ~ 30 kDa (M23-AQP4) and ~ 32 kDa (M1-AQP4) in WT and as one band at ~ 32 kDa (M1-AQP4) in OAP-null astrocyte protein samples. Refer to Figure S3 for uncropped western blots of AQP4. (**b**) Densitometric analysis of the differential expression of M1-AQP4 and M23-AQP4) isoforms normalized against GAPDH for in WT astrocytes and M1-AQP4 isoform expression normalized against total protein in OAP-null astrocytes. Mann–Whitney test, N = 5 independent experimental dataset, *n* = 10 per each group (*p < 0.05, **p < 0.001, ***p < 0.001). Both isoforms undergo a statistical significant decrease in IL-1β/TNF-α treated astrocytes compared to CTRL in both genotypes. (**c**) Normalized (F/F0) water transport kinetics of calcein-AM loaded astrocytes upon 60 mOsm/L hypotonic gradient at 20 °C. Curves are representative of N = 3 independent experimental dataset, with *n* ranging from 28 to 41. (**d**) Box plot showing the fold change of time constants (τ) of cell swelling. Data highlights no significant changes in cell swelling rate upon hypoosmotic challenge in astrocytes exposed to pro-inflammatory stimuli compared to their controls despite a profound change in AQP4 expression. Two-way ANOVA with Tukey’s Multiple Comparison test. Refer to Table S5 for details on significant differences between groups and related *p-*values. (**e**) Box plot showing the amplitude of cell swelling which is exclusively increased in IL-1β/TNF-α–treated OAP-null cells compared to their controls. Two-way ANOVA with Tukey’s Multiple Comparison test. Refer to Table S6 for details on significant differences between groups and related *p-*values.

We next measured to what extent the functional impairment of collective migration in inflamed astrocytes was associated with changes in AQP4 expression and AQP4-dependent water transport (Fig. 6).

Western blot analysis revealed that IL-1β/TNF-α exposure significantly reduced both AQP4 isoforms, M1 and M23, levels in both WT and OAP-null astrocytes. WT cells expressed both M1- and M23-AQP4 isoforms, whereas OAP-null astrocytes only expressed the M1-AQP4 isoform, as expected. Surprisingly, despite the marked reduction in AQP4 protein levels, IL-1β/TNF-α treatment did not significantly affect swelling kinetics in either genotype. Interestingly, inflamed OAP-null astrocytes exhibited a greater swelling amplitude, suggesting increased passive volume uptake. These results indicate that while AQP4 abundance correlates with water permeability in untreated cells, inflammation uncouples this relationship, likely by altering cell morphology or increasing surface-to-volume ratios.

### IL-1β/TNF-α treatment strongly impairs gap-junctional connectivity by reducing Cx43 expression and plaque formation

**Fig. 7:**
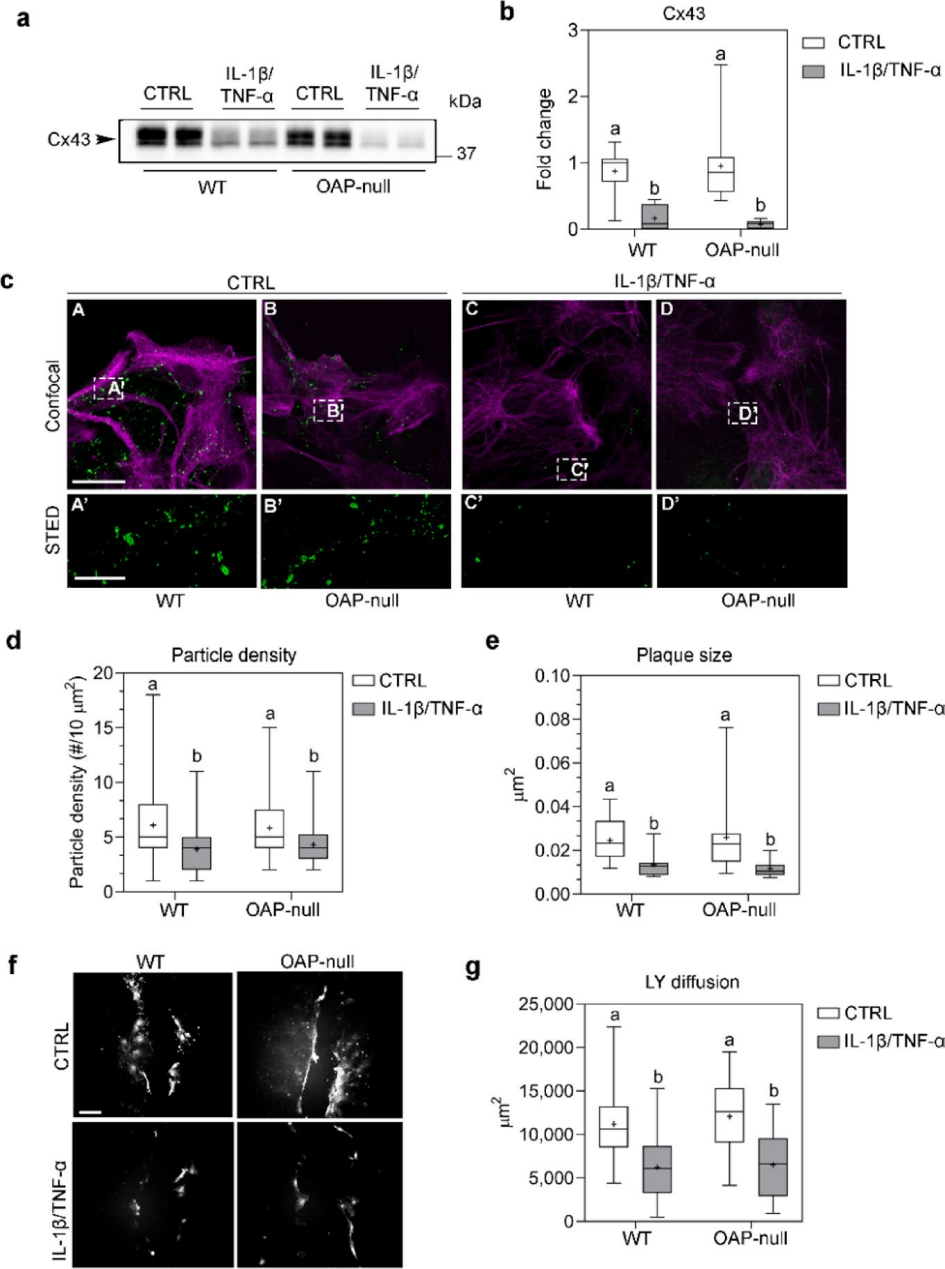
Cx43 expression and gap junction-regulated astrocyte connectivity. (**a**) Western Blot of Cx43 expression revealed as two bands at ~ 40 kDa. Refer to Figure S4 for uncropped western blot of Cx43. (**b**) Densitometric analysis of Cx43 normalized against total protein. Data are expressed as mean ± SEM. Two-way ANOVA with Tukey’s Multiple Comparison test, N = 5 independent experimental dataset, *n* = 10 for each group. Refer to Table S7 for details on significant differences between groups and related *p-*values. **(c)** Confocal images of GFAP (magenta) and Cx43 (green) staining (A-D; scale bar: 25 µm). The white boxes indicate the regions imaged by STED microscopy (E–H; scale bar: 5 µm) showing the Cx43-based intercellular junctions. (**d**) Box plot showing Cx43-particle density measured as the number of STED-imaged Cx43-plaques within a 10 µm^2^ region. The particle size is significantly decreased in IL-1β/TNF-α-treated astrocytes compared to CTRL cells. Two-way ANOVA with Tukey’s Multiple Comparison test, with *n* ranging from 14 to 29 fields from N = 3 independent experimental dataset. Refer to Table S8 for details on significant differences between groups and related *p-*values. (**e**) Box plot showing the size of Cx43-plaques which is greatly reduced in IL-1β/TNF-α-treated astrocytes with respect to controls. Two-way ANOVA with Tukey’s Multiple Comparison test, *n* ranging from 14 to 29 fields from N = 3 independent experimental dataset. Refer to Table S9 for details on significant differences between groups and related *p-*values. (**f**) Gap junctional intercellular communication assessed by Lucifer Yellow (LY) scrape loading assay (scale bar = 100 µm). (**g**) Quantification of LY-spreading across the cell sheet expressed as stained area (µm^2^). Two-way ANOVA with Tukey’s Multiple Comparison test, with *n* ranging from 40 to 74 fields from N = 2 independent experimental dataset. Refer to Table S10 for details on significant differences between groups and related *p-*values.

Given the key role of gap junctions in coordinating astrocyte networks, we evaluated the expression and spatial organization of Connexin-43 (Cx43) and assessed the functional consequences of IL-1β/TNF-α treatment on intercellular communication (Fig. 7).

Western blot analysis revealed a dramatic downregulation of Cx43 in IL-1β/TNF-α-treated astrocytes of both genotypes, with no detectable differences between WT and OAP-null under control conditions. Super-resolution STED imaging confirmed that in untreated astrocytes, Cx43 formed plaques of heterogeneous sizes and densities uniformly across cell–cell junctions. In contrast, inflammatory treatment led to a substantial reduction in plaque size and particle density, independently of AQP4 aggregation state. These structural changes were mirrored by functional alterations in gap-junctional communication. The Lucifer Yellow scrape-loading assay revealed robust and comparable dye spread across the monolayer in CTRL WT and OAP-null astrocytes, whereas treated cultures of both genotypes showed a marked reduction in dye transfer. Together, these data demonstrate that chronic cytokine exposure severely impairs gap junction assembly and function, likely contributing to the mechanical discoordination and loss of collective motility observed under inflammatory conditions, independently of AQP4 clustering.

## Discussion

This study demonstrates that AQP4-tetramers natively expressed in primary cultured astrocytes facilitate cell migration, in contrast to the current literature, which has so far focused on immortalized cell lines, or on astrocytes transfected with recombinant AQP4 constructs^26^. Furthermore, while earlier work provided valuable insights into the molecular and biomechanical mechanisms in the movement of individual cells^26^, our study demonstrated that AQP4-tetramers enhance the collective behavior of migrating cells. The work also shed light on the features and putative mechanisms that govern their coordinated motion, thus deciphering a new functional role of the AQP4-aggregation state in the physiology and mechanobiology of glial cells. The main findings of this study also rely on providing new insights into astrogliosis and astrocytes response to chronic pro-inflammatory stress in vitro.

The pivotal result of our study shows that under control conditions the selective and native expression of AQP4-tetramers (as in OAP-null cells) enhances the migratory performances of cellular sheets in wound healing experiments to a greater extent than AQP4-OAPs (as in WT cells). Conversely, pro-inflammatory treatment completely disrupts astrocytes ability to heal the wound, as previously found in similar conditions^46^, remaining either largely immobile or slowly migrating in the direction opposite to the scratch.

Previous findings obtained in M1-AQP4-transfected cell lines suggest that enhanced migration occurs because small tetramers can diffuse more efficiently than OAPs toward the lamellipodium^26^ where AQP4-mediated water flows can generate pushing forces for cellular movement, as proposed for aquaporins in general^12,15^. However, since lamellipodia are predominantly found at the leading edge of migrating cell sheet, we hypothesize that AQP4 might play a role beyond lamellipodium formation.

The immunostaining of F-actin revealed expansive lamellipodia and filopodia in control migrating astrocytes at the front end (“leader cells”) of both genotypes, with WT displaying more filopodia than OAP-null cells. Conversely, astrocytes treated with IL-1β/TNF-α resulted to be completely lacking lamellipodia but displaying cytoplasmic stress fibers.

In migrating cell sheets, a leader–follower cell hierarchy is established to enable a fine force distribution throughout the bulk and sustain a collective forward movement^47^. The analysis of AQP4 and F-actin labeling in the follower cells revealed interesting insights on the effect of the treatment, especially in cytoskeleton rearrangements, explaining some of the differences observed in the collective motion in the four conditions. In particular, control and reactive cells showed a marked difference in the organization of actin cortical fibers along the lateral edges of follower cells, but no net nor preferential distribution of AQP4 throughout the bulk in leader *versus* follower cells.

In the model proposed by Friedl et al.^48^, the leader cells in the front are mechano-coupled to follower cells through supracellular actomyosin-based structures via small GTPases RhoA activity, which ensures longitudinal and transversal force transmission. More specifically, it has been suggested that while in leader cells cytoskeleton fibers rearrange predominantly in protrusive lamellipodia, in the follower cells cortical actomyosin fibers (or “actin belt”^47^) rim the edges of the cells to disable the formation of cell protrusions and mechanically stabilize the whole group.

Our evidence suggests that control astrocytes preserve an intact actin belt in the follower cells that likely sustains forward migration and enables the even distribution of longitudinal and transversal forces across the cell monolayer. Instead, IL-1β/TNF-α treatment appears to disrupt the cortical actomyosin network in both leader but especially follower cells, thus abolishing or reversing the direction of motion by putatively impairing or inhibiting force transmission. Interestingly, the formation of stress fibers and the disassembly of F-actin structures were previously observed in other cell types under similar experimental conditions^49^. Hence, we here propose the actomyosin belt preservation/breakdown as the mechanistic rationale for the forward/impaired movement observed in ‘migrating’ control and ‘non-migrating’ IL-1β/TNF-α-treated astrocytes (Fig. 5).

AQP4 western blot assays revealed that IL-1β/TNF-α-treated cells showed a significant reduction in AQP4 global expression levels compared to their controls. However, even if cell swelling rates were found to be slower in OAP-null astrocytes compared to WT under control conditions, as previously reported^24^, treated cells, which downregulate AQP4 expression levels, showed similar water transport rates compared to their controls, unexpectedly.

These results provided information at multiple levels. Firstly, they rule out that collective migration is merely influenced by the molecular levels of AQP4 and the average water membrane rates, since they both resulted to be lower in the most migrating phenotype, the OAP-null. It is likely, instead, that the intrinsic ability of astrocytes to undergo cell swelling upon hypotonic stimulus does not linearly reflect fine changes in water permeability that might still occur in microdomains at the leading edge of migrating cells and can even remain preserved in the absence of AQP4^36,50^. We here propose that differences in water permeability per se are unlikely to explain the observed migratory phenotypes, and that rather than bulk water transport, AQP4 contribution to astrocyte motility involves structural and mechanotransductive functions. This reinforces the concept that AQP4–cytoskeleton interactions, and their consequent effects on cellular mechanics, adhesion, and polarity, are the key determinants of astrocyte migration^25,51–53^, and suggests that AQP4, through its supramolecular organization and linkage to the cytoskeletal scaffold, may also influence how astrocytes sense and respond to mechanical cues within their microenvironment.

Secondly, they provided new insights into water transport and cell volume variations dependent on AQP4-aggregation state and treatment. When AQP4 is organized into OAPs, its abundance enhances water transport by providing a sufficient number of pores for water exchange, differently from OAP-null cells, where AQP4 tetramers are functional yet reduced^24^ and even lower upon inflammation. However, we here hypothesize that even with fewer OAPs, as in IL-1β/TNF-α-treated WT cells, astrocytes may still provide a sufficient total number of AQP4 channels required for rapid water transport and effective osmotic equilibration.

Besides aquaporins, connexins are known to play a key role in cell migration by forming a molecularly and functionally coordinated syncytial network, which regulates astrocytes’ communication^54^. As no significant differences were observed between control WT and OAP-null astrocytes, results highlight that GJ connectivity and Cx43-dependent communication are physiologically triggered in migrating cells to the same extent regardless of AQP4 supra molecular level of aggregation, but dramatically impaired in treatment conditions, reinforcing its essential role in facilitating cell migration. However, we exclude the possibility that Cx43 differentially cooperates with AQP4 tetramers to regulate or boost the motion of OAP-null astrocyte sheets. This suggests that other compensatory mechanisms or signaling pathways may sustain the enhanced migration observed in OAP-null astrocytes, independently of direct Cx43-AQP4 interplay under control conditions.

The quantification and extraction of large-scale bulk movements have been quantified extensively in several collective motion datasets, such as in tumoral^55^ and epithelial cell migration^56^, bacterial lattices^57^, and cell turbulence^58^. The PIV analysis revealed that cells under control conditions feature the distinct ability to move predominantly horizontally. This suggests that they efficiently orient and direct their collective movement toward the same final destination (i.e. the open space of the wound) likely through the AQP4 physiological expression and polarization and preservation of intact actin belt, both impaired in IL-1β/TNF-α-treated astrocytes, which express atypical or very low levels of aquaporins.

**Fig. 8:**
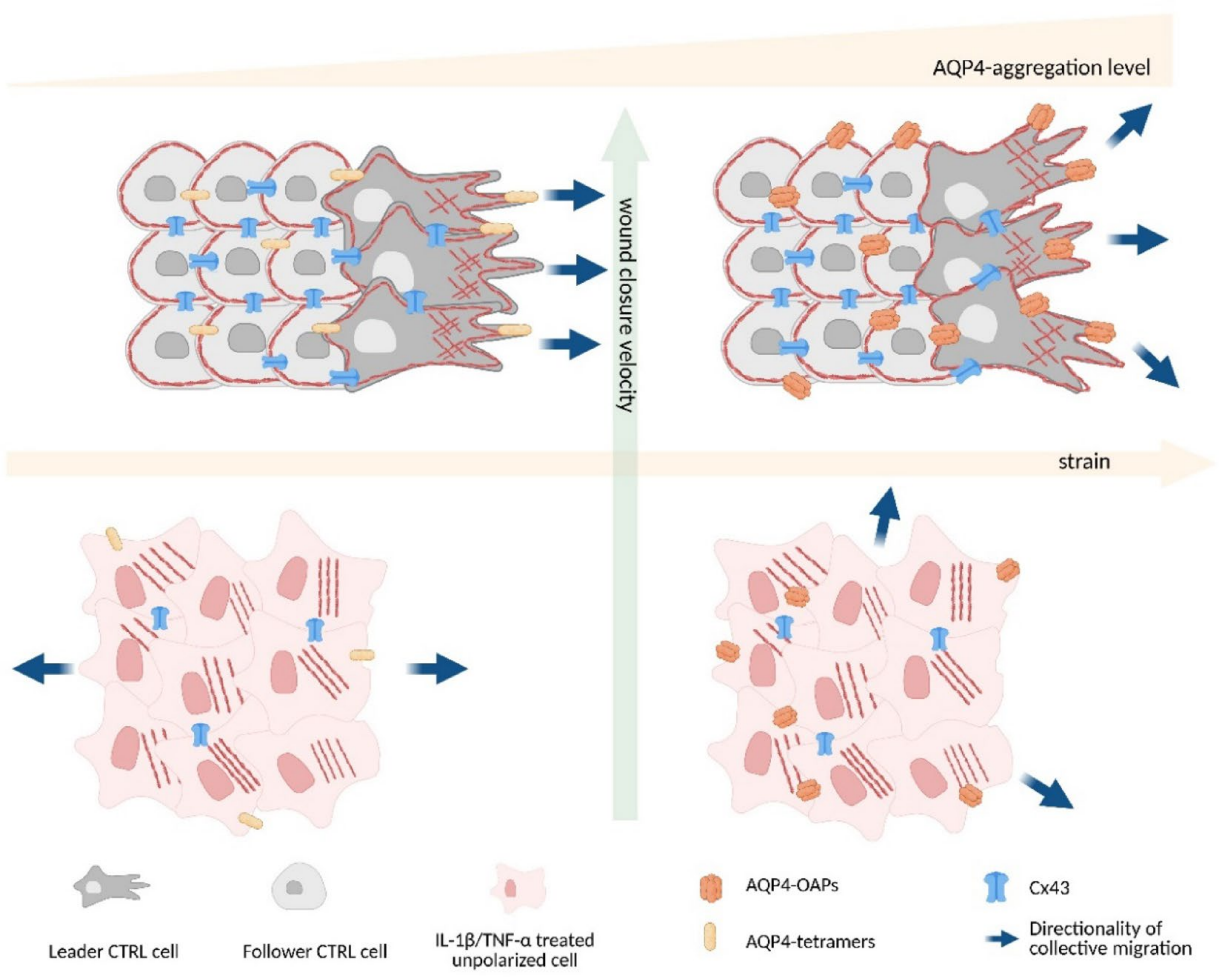
Proposed model of collective migration in OAP-null and WT astrocytes under control and treatment conditions (Created in BioRender. Barile, B. (2026) https://BioRender.com/8cdvcse).

On the other hand, the analysis of cellular strain dissected a more precise contribution of AQP4-tetramers, demonstrating their ability to suppress intercellular repulsion and promote a more uniform, straight and coordinated collective migration pattern, accelerating their arrival compared to WT. We therefore hypothesize that OAP-null sheets are pulled more coherently by stabilizing the cytoskeletal organization and minimizing directional fluctuations (Fig. 8).

Interestingly, the hypothesis that AQPs can improve cell migration directionality by facilitating lamellipodium formation towards the chemotactic gradient, enabling the cell to migrate along a less tortuous path, was proposed long ago^59^. However, to the best of our knowledge, this is the first study showing the role of AQP4 in speed, directionality, and tortuosity in the collective guidance.

How the lack of OAPs causes this increased coordination is still unclear and deserves in-depth future analysis. However, we foresee a physiological meaning. Rather than a loss of the collective behavior, we anticipate that the increased strain might be a beneficial feature in vivo used by the cells to facilitate a more erratic but exploratory pattern in their movement to respond to a variety of extracellular chemical and mechanical cues or even prevent tumoral progression or transformation, disfavoring uncontrolled migration and locomotion stop signals escaping.

Reactive astrogliosis is detected in nearly all brain injuries and neurological disorders, especially under the simulation of cytokines and inflammatory mediators produced by traumas^11^.

The data shown in this study illustrate that IL-1β/TNF-α chronic stimulation generates a reactive astrocytic phenotype with reduced connectivity and migratory ability. In a recent study, Banitalebi and colleagues found reduced density (or total absence) of AQP4 in the core of experimentally induced ischemic infarcts and a mislocalization of AQP4 assemblies in the borders of the scar^60^. In light of this, we speculate that AQP4 downregulation might occur upon the prolonged exposure of astrocytes to cytokines occurring either artificially in vitro or in vivo within the lesion core of ischemic infarcts as a side/adverse effect of chronic inflammation. On the other hand, its upregulation or positioning might be promoted in later stages of glial scar deposition or in differently located subpopulation of astrocytes exposed to different stimuli (i.e., anti-inflammatory; chemotactic).

In conclusion, our study has uncovered a novel physiological role of AQP4 and its supramolecular aggregation in collective cell migration. Specifically, we demonstrated that the non-aggregated state of AQP4 enhances cell motility by tuning directionality and reducing cell strain, without affecting intercellular communication mediated by connexins. Instead, connexin-mediated communication is disrupted under chronic exposure to proinflammatory cytokines, which also alters cytoskeletal organization, cell polarity, and AQP4 molecular levels.

From a broader perspective, our findings provide valuable insights into cell migration dynamics occurring at the collective level. Given the crucial role of AQP4 in astrocyte function, understanding how its aggregation state influences cell behavior may have significant implications for human health, including brain development, tissue repair, and cancer metastasis.

The main limitation of this study is ascribable to the use of primary astrocytes which may differ from their in vivo counterparts due to the absence of cell–cell contacts, vasculature, and extracellular matrix, factors that could alter their responses compared to astrocytes in the intact brain^61,62^. Moreover, cultured astrocytes typically lose the complex, highly branched morphology and microdomain organization that characterize their polarized architecture in vivo^61,63^. Consequently, AQP4 expression becomes uniformly distributed along the plasma membrane, leading to a marked reduction in OAP formation^37,64^. Despite these limitations, the use of primary astrocyte cultures from OAP-null mice enabled us to directly assess the intrinsic contribution of AQP4 supramolecular organization to astrocyte dynamics, independently of tissue-level specialization. The advantage of this culture system provided the possibility to perform experiments under highly controlled conditions, helping us to answer the question as to whether or not the importance of OAP organization is directly associated with astrocyte migratory behavior^26^. Another limitation of this work lies in the fact that we have not yet uncovered the mechanistic basis underlying the observed differences in collective guidance. While we demonstrated that astrocyte behavior varies depending on both the AQP4 aggregation state and the presence of inflammation, the precise molecular and biophysical mechanisms driving these distinct migratory phenotypes remain unclear. Further investigation is needed to dissect how AQP4 organization and inflammatory cues converge to influence collective dynamics and directional guidance in astrocyte populations.

## Supplementary Information

Below is the link to the electronic supplementary material.

Supplementary Material 1

Supplementary Material 2

## Data Availability

The data that support the findings of this study are available from the corresponding author upon reasonable request.

## Declarations

### Statements

*Grammarly* has been used to correct spelling, grammar, and punctuation and polish English language. Authors thank Gaetano Devito for animal care. The manuscript is original, it has not been published nor is under consideration for publication elsewhere. All authors have approved its submission. Care of experimental animals was in accordance with institutional guidelines (F2AEC.N.ETU). The data that support the findings of this study are available from the corresponding author upon reasonable request.
