# Supplementary material for: Supramolecular aggregation of aquaporin-4 shapes astrocyte collective migration and mechanics
Source: Sci Rep. 2026 Jan 22;16:6021. doi: 10.1038/s41598-026-35900-z (PMC12901288; doi:10.1038/s41598-026-35900-z)
Supplement: Supplementary file 1 — Supplementary Material 1 [file 41598_2026_35900_MOESM1_ESM.docx]

**Figure S1. Effects of FBS concentrations in cell migration assays and cell metabolic activity in primary cultured astrocytes grown in 10% FBS growth medium for 8 days (“WT 10% FBS”) and in 10% FBS growth medium for 7 days followed by 1% FBS growth medium for 1 day (“WT 1% FBS”)**. (**a**) Box plots showing wound healing percentages kinetics of the scratch wound closure. Multiple unpaired t-test was performed on the mean values between the two groups for each time point (N=3, n=28 for WT astrocytes +10% FBS; N=3, n=44 for WT astrocytes +1% FBS). (**b**) Box plots showing normalized metabolic activity of WT astrocytes cultured in media containing either 10% FBS or 1% FBS. Briefly, astrocytes were maintained for 7 days in 10% FBS growth medium, or for 6 days in 10% FBS followed by 1 day in 1% FBS before performing the MTT assay. Unpaired t-test was performed on the mean values between the two groups (N=2, n=8).


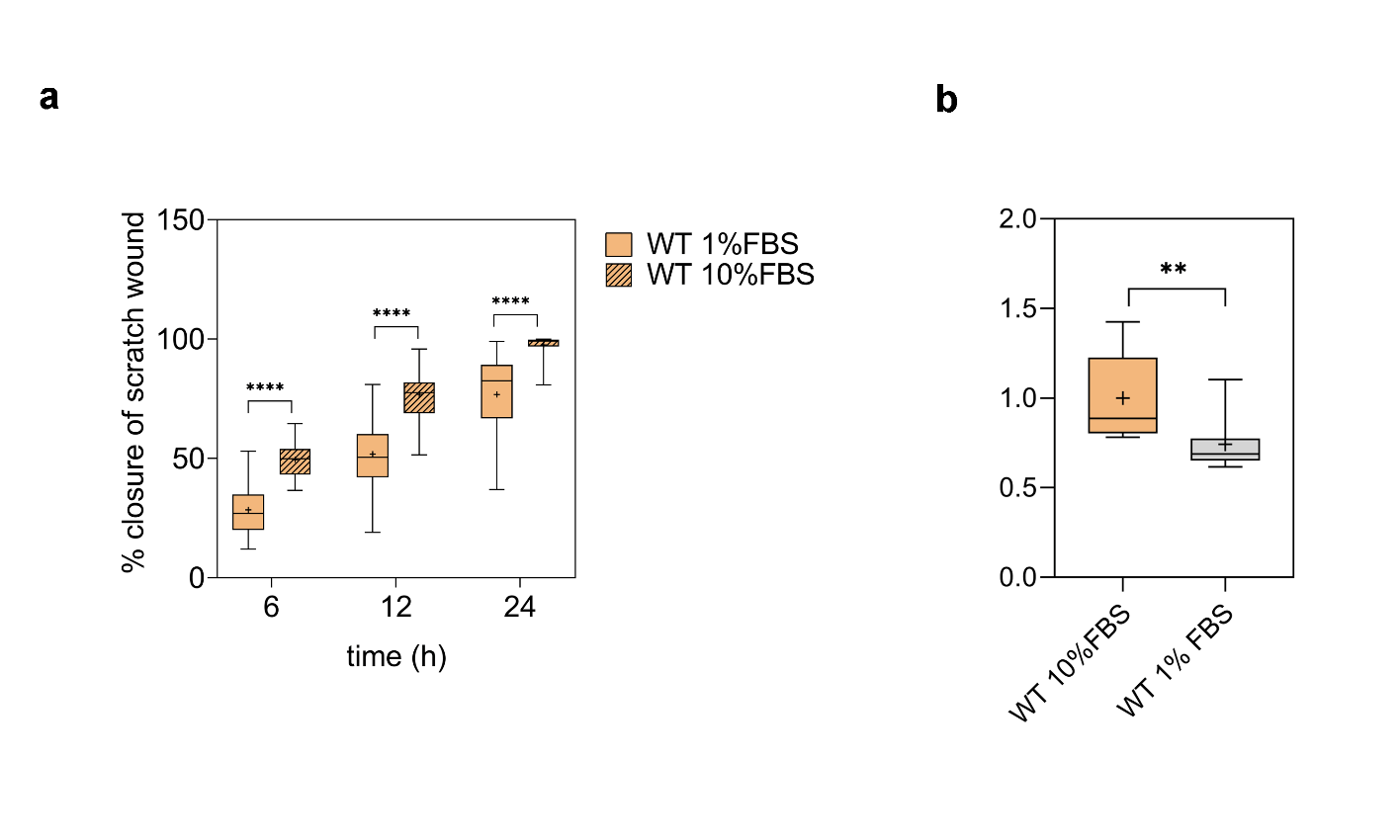


**Figure S2. Uncropped western blot image of GFAP from Figure 1a.**

**
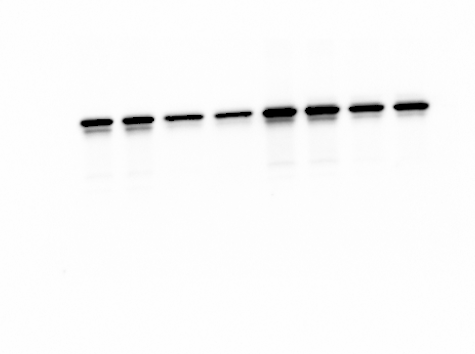
**

**Figure S3. Uncropped western blot images of AQP4 (left) and resolved AQP4 isoforms AQP4-M1 and AQP4-M23 (right) from Figure 6a.**

**
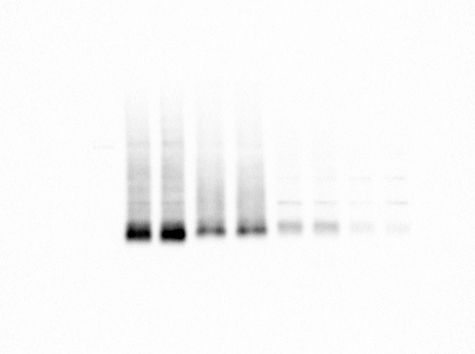
**
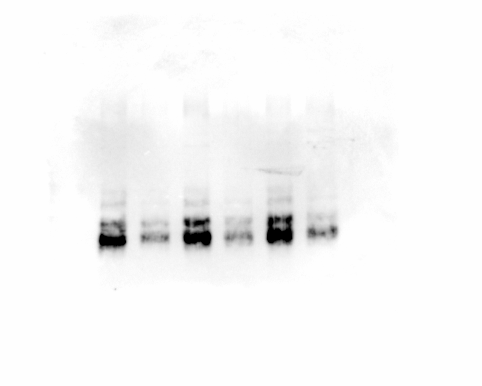


**Figure S4. Uncropped western blot image of Cx43 from Figure 7a.**

**
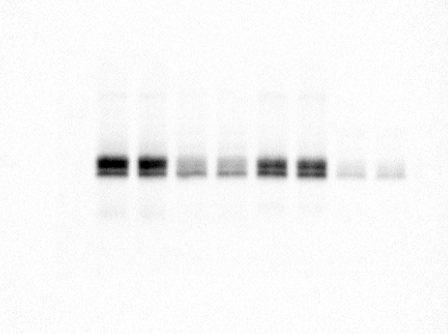
**
