# Supplementary material for: Supramolecular aggregation of aquaporin-4 shapes astrocyte collective migration and mechanics
Source: Sci Rep. 2026 Jan 22;16:6021. doi: 10.1038/s41598-026-35900-z (PMC12901288; doi:10.1038/s41598-026-35900-z)
Supplement: Supplementary file 2 — Supplementary Material 2 [file 41598_2026_35900_MOESM2_ESM.docx]

**Table S1.**

Two-way ANOVA with Tukey’s Multiple Comparison test for GFAP expression levels.

| WT CTRL vs. WT IL-1β/TNF-α | *** |
| --- | --- |
| WT CTRL vs. OAP-null CTRL | ns |
| WT CTRL vs. OAP-null IL-1β/TNF-α | ** |
| WT IL-1β/TNF-α vs. OAP-null CTRL | ** |
| WT IL-1β/TNF-α vs. OAP-null IL-1β/TNF-α | ns |
| OAP-null CTRL vs. OAP-null IL-1β/TNF-α | ** |

Table S2.

Two-way ANOVA with Tukey’s Multiple Comparison test for GFAP^+^ area expressed in µm^2^.

| WT CTRL vs. WT IL-1β/TNF-α | * |
| --- | --- |
| WT CTRL vs. OAP-null CTRL | ns |
| WT CTRL vs. OAP-null IL-1β/TNF-α | * |
| WT IL-1β/TNF-α vs. OAP-null CTRL | * |
| WT IL-1β/TNF-α vs. OAP-null IL-1β/TNF-α | ns |
| OAP-null CTRL vs. OAP-null IL-1β/TNF-α | ** |

**Table S3.**

Two-way ANOVA with Tukey’s Multiple Comparison test for filopodia length.

| WT CTRL vs. OAP-null CTRL | ns |
| --- | --- |
| WT CTRL vs. WT IL-1β/TNF-α | **** |
| WT CTRL vs. OAP-null IL-1β/TNF-α | * |
| OAP-null CTRL vs. WT IL-1β/TNF-α | **** |
| OAP-null CTRL vs. OAP-null IL-1β/TNF-α | * |
| WT IL-1β/TNF-α vs. OAP-null IL-1β/TNF-α | ns |

**Table S4.**

Two-way ANOVA with Tukey’s Multiple Comparison test for filopodia density.

| WT CTRL vs. OAP-null CTRL | *** |
| --- | --- |
| WT CTRL vs. WT IL-1β/TNF-α | * |
| WT CTRL vs. OAP-null IL-1β/TNF-α | *** |
| OAP null ctrl vs. WT IL-1β/TNF-α | ns |
| OAP-null CTRL vs. OAP-null IL-1β/TNF-α | ns |
| WT IL-1β/TNF-α vs. OAP-null IL-1β/TNF-α | ns |

Table S5.

Two-way ANOVA with Tukey’s Multiple Comparison test of cell swelling time constants (τ) fold change.

| WT CTRL vs. WT IL-1β/TNF-α | ns |
| --- | --- |
| WT CTRL vs. OAP-null CTRL | *** |
| WT CTRL vs. OAP-null IL-1β/TNF-α | **** |
| WT IL-1β/TNF-α vs. OAP-null CTRL | ** |
| WT IL-1β/TNF-α vs. OAP-null IL-1β/TNF-α | **** |
| OAP-null CTRL vs. OAP-null IL-1β/TNF-α | ns |

Table S6.

Two-way ANOVA with Tukey’s Multiple Comparison test of swelling amplitude.

| WT CTRL vs. WT IL-1β/TNF-α | ns |
| --- | --- |
| WT CTRL vs. OAP-null CTRL | ns |
| WT CTRL vs. OAP-null IL-1β/TNF-α | ns |
| WT IL-1β/TNF-α vs. OAP-null CTRL | ** |
| WT IL-1β/TNF-αvs. OAP-null IL-1β/TNF-α | ns |
| OAP-null CTRL vs. OAP-null IL-1β/TNF-α | **** |

Table S7.

Two-way ANOVA with Tukey’s Multiple Comparison test for Cx43 expression levels.

| WT CTRL vs. WT IL-1β/TNF-α | *** |
| --- | --- |
| WT CTRL vs. OAP-null CTRL | ns |
| WT CTRL vs. OAP-null IL-1β/TNF-α | *** |
| WT IL-1β/TNF-α vs. OAP-null CTRL | *** |
| WT IL-1β/TNF-α vs. OAP-null IL-1β/TNF-α | ns |
| OAP-null CTRL vs. OAP-null IL-1β/TNF-α | **** |

Table S8.

Two-way ANOVA with Tukey’s Multiple Comparison test for Cx43 density.

| WT CTRL vs. WT IL-1β/TNF-α | *** |
| --- | --- |
| WT CTRL vs. OAP-null CTRL | ns |
| WT CTRL vs. OAP-null IL-1β/TNF-α | ** |
| WT IL-1β/TNF-α vs. OAP-null CTRL | *** |
| WT IL-1β/TNF-α vs. OAP-null IL-1β/TNF-α | ns |
| OAP-null CTRL vs. OAP-null IL-1β/TNF-α | ** |

Table S9.

Two-way ANOVA with Tukey’s Multiple Comparison test for Cx43 size.

| WT CTRL vs. WT IL-1β/TNF-α | * |
| --- | --- |
| WT CTRL vs. OAP-null CTRL | ns |
| WT CTRL vs. OAP-null IL-1β/TNF-α | ** |
| WT IL-1β/TNF-α vs. OAP-null CTRL | ** |
| WT IL-1β/TNF-α vs. OAP-null IL-1β/TNF-α | ns |
| OAP-null CTRL vs. OAP-null IL-1β/TNF-α | *** |

Table S10.

Two-way ANOVA with Tukey’s Multiple Comparison test for LY spreading across cell sheets.

| WT CTRL vs. WT IL-1β/TNF-α | **** |
| --- | --- |
| WT CTRL vs. OAP-null CTRL | ns |
| WT CTRL vs. OAP-null IL-1β/TNF-α | **** |
| WT IL-1β/TNF-α vs. OAP-null CTRL | **** |
| WT IL-1β/TNF-α vs. OAP-null IL-1β/TNF-α | ns |
| OAP-null CTRL vs. OAP-null IL-1β/TNF-α | **** |
